# Welcome to *Neural Systems and Circuits*: bridging the gap between theory and experiment

**DOI:** 10.1186/2042-1001-1-1

**Published:** 2011-01-26

**Authors:** Peter E Latham, Venkatesh N Murthy

**Affiliations:** 1 Gatsby Computational Neuroscience Unit, UCL, London, UK; 2 Department of Molecular and Cellular Biology, and Center for Brain Science, Harvard University, Cambridge, MA, USA

## 

Neuroscientists study the brain at many levels of organization - from single molecules to the entire brain. Over the past 30 odd years, advances in molecular biology, genetics and biophysical methods have revolutionized studies at the small scale - ion channels, synapses and subcellular compartments. At the other end, advances in macroscopic brain mapping have ignited a wave of studies at the large scale. However, the computations carried out by brains are best understood in terms of the collective behavior of neuronal populations. Although we have known the value of circuit-level analysis for some time now, limitations in experimental and analytical tools have been significant barriers for progress. Brains are made up of staggering numbers of neurons (~10^5 ^in fruit flies, ~10^11 ^neurons in humans); we don't know the detailed connectivity, we don't have a detailed understanding of the input and output, and we don't know the algorithms. We are often probing in the dark.

Things are starting to change, and mesoscale studies involving circuits of neurons are now ready to take centre stage. Optical and genetic techniques now allow us to visualize and perturb activity of identified populations of neurons at the microscopic scale; serial electron microscopy and clever molecular circuit-tracing techniques are on the verge of giving us detailed information on connectivity among large numbers of neurons; and theoretical neuroscientists have embraced the messiness of biological hardware and started to think about how networks actually compute.

These new tools and techniques are poised to produce a torrent of new experimental results and theoretical insights. *Neural Systems & Circuits *will offer a forum for the seamless integration of the resulting body of knowledge. We welcome both theoretical and experimental studies, and everything in between, as long as they relate to circuit or systems-level analysis. In fact, we anticipate an increasing number of these "in between" studies, as the boundaries between theory and experiment blur. Too often, theoretical papers become mathematical exercises for specialists, with little attempt to engage or stimulate experimentalists (who in turn, simply ignore most theory papers). Conversely, many experimental studies read like catalogues, offering little incentive to others wanting to integrate them into a bigger picture or a theory. By offering a mix of papers in a single locus, we aim to foster communication among theorists and experimentalists, and so catalyze cross-pollination.

*Neural Systems & Circuits *is a fully open-access journal. Articles will be published online as soon as they are accepted, and it is anticipated that they will also be indexed in PubMed in due course. Besides research articles, we will publish reviews and opinion pieces. We hope to report, on occasion, dialogs between those with opposing points of view on important and controversial topics. Studies of disease that have particular emphasis on circuits are also welcome, as are technical advances. The diverse and expert editorial board will ensure excellence in the review process.

This inaugural issue of *Neural Systems & Circuits *presents a selection of articles on different topics and aims to illustrate some of the topics we would like to include as the journal grows. As our understanding of the field changes, we expect the journal to grow to accommodate the latest advances. We are committed to making *Neural Systems & Circuits *a success, and sincerely hope that you will share our vision and will consider contributing to this new journal.

